# Rates of protein synthesis are reduced in peripheral blood mononuclear cells (PBMCs) from fragile X individuals

**DOI:** 10.1371/journal.pone.0251367

**Published:** 2021-05-11

**Authors:** Olivier Dionne, Audrey Lortie, Florence Gagnon, François Corbin

**Affiliations:** Department of Biochemistry and Functional Genomic, Faculty of Medicine and Health Sciences, Université de Sherbrooke and Centre de Recherche du CHUS, CIUSSS de l’Estrie-CHUS, Sherbrooke, Quebec, Canada; Centre National de la Recherche Scientifique, FRANCE

## Abstract

**Background:**

Fragile X syndrome (FXS) is the leading inherited cause of intellectual disability and is caused by the loss of expression of the Fragile X mental retardation protein (FMRP). In animal model of FXS, the absence of FMRP leads to an aberrant rate of neuronal protein synthesis, which in turn is believed to be at the origin of defects regarding spine morphology and synaptic plasticity. Normalisation of protein synthesis in these models has been associated with a rescue of FXS behavioral and biochemicals phenotype, thus establishing the rate of protein synthesis as one of the most promising monitoring biomarker for FXS. However, rate of protein synthesis alteration in fragile X individuals is not well characterized.

**Method:**

We applied a robust radiolabeled assay to measure rate of protein synthesis in freshly extracted peripheral blood mononuclear cells (PBMCs) and blood platelets. We ultimately settle on PBMCs to measure and compare rate of protein synthesis in 13 males with fragile X and 14 matched controls individuals.

**Results:**

Using this method, we measured a 26.9% decrease (p = 0,0193) in the rate of protein synthesis in fragile X individuals PBMCs. Furthermore, the rate of protein synthesis measurements obtained were highly reproducible, highlighting the robustness of the method.

**Conclusion:**

Our work presents the first evidence of a diminution of the rate of protein synthesis in a human peripheral model of fragile X. Our results also support the finding of previous studies using brain PET imaging in Fragile X individuals. Since our assay only requires a simple venous puncture, it could be used in other cases of intellectual disability in order to determine if an aberrant rate of protein synthesis is a common general mechanism leading to impairment in synaptic plasticity and to intellectual disability.

## Introduction

Intellectual disability (ID) is a developmental condition characterized by significant deficit in intellectual functioning (IQ score below 70) and adaptive behaviour affecting approximately 1% of males. Fragile X syndrome (FXS) is the most common monogenic cause of intellectual disabilities (ID) affecting 1 per 5000 males [1]. Although variable, most FX males have moderate to severe ID (IQ < 55). Aside from cognitive dysfunctions, fragile X (FX) individuals are prone to develop a wide range of medical and behavioral problems [2, 3]. The vast majority (> 99%) of FX cases arise from the runaway expansion of CGG repeats found in the 5’ UTR of the *FMR1* gene and by the subsequent methylation of CpG islands in its promoter. This phenomenon ultimately results in the transcriptional silencing of *FMR1* and subsequent loss of expression of the Fragile X mental retardation protein (FMRP) [4–6]. FMRP is well-conserved across species and expressed in nearly every cells or tissues, including lymphocytes and platelets [7, 8]. FMRP is a RNA-binding protein mainly associated with polyribosomes [9] that would mainly acts as a translational regulator of its targeted mRNAs. Previous studies have reported that FMRP binds approximately 4% of brain mRNA, a subset likely to be essential for the regulation of synaptic plasticity and spine morphogenesis [10–12]. Consequently, the absence of FMRP, and its concomitant effect on translational regulation of this subset of mRNA, would cause the cognitive and behavioral impairments found in FXS

The generation of the *Fmr1* KO mouse allowed the investigation of the impact of FMRP absence on brain’s protein synthesis. Utilizing this model, in conjunction with different *in vitro*, *ex vivo* and *in vivo* experimental techniques, several groups have reported a FMRP-dependant increase in the global rate of protein synthesis [13–16]. Moreover, its normalisation using targeted drugs has been associated with the rescue of the FX phenotype; bringing dysregulation of the rate of protein synthesis as a cornerstone of FX physiopathology and as a potential objective outcome measure for FXS clinical trials [17–22]. However, this global increase in protein synthesis do not completely reflects the impact of FMRP’s absence on mRNA translation, as some proteins are also found downregulated or not affected in such context [23]. Furthermore, it’s still unclear if the net increase in protein synthesis found in KO mice brains is present in every developmental stage, tissue or even in FX individuals.

**Characterizing brain’s rate of protein synthesis non-invasively in human is not an easy task**. **As such**, only two studies have addressed this issue in FXS individuals using positron emission tomography (PET) imaging. The first report concluded that the rate of protein synthesis is lower in FXS brains subjected to propofol sedation [24]. Although not statistically significant, the same group recently reported a similar effect using dexmedetomidine as a sedating agent instead of propofol; thereby strongly suggesting that the observed decreased in the rate of protein synthesis is not caused by sedating agents [25]. These reports shed new light on translational regulation in FXS and highlights limitations of the Fmr1 KO mouse model to fully replicate FXS phenotype and the necessity to further study FX subjects in order to get a better understanding of the human physiopathology [26]. Such task represents a substantial challenge due to the inaccessibility and invasive nature of neuronal tissue, thus emphasizing the need to use a less invasive model.

We and others have previously shown that peripheral blood cells are great surrogate model for studying mechanisms underlaying FXS physiopathology and clinical response. Indeed, both PBMCs (peripheral blood mononuclear cells) [27–30] and platelets [31–34] have shown to reproduce some of the core biochemical phenotypes of FX neurons. Furthermore, blood cells collection is minimally invasive and can be achieved repeatedly, making them ideal for clinical trials [33]. However, rate of protein synthesis in these cells has not been well studied.

The goal of this study was to determine, using freshly isolated peripheral blood cells (PBMCs and platelets), if the rate of protein synthesis is either increased or decreased in FXS patients as compared to matched controls. In order to increase our chance to observe a statistical difference in the rate of protein synthesis between FX individuals and healthy controls, only fully mutated FX males were recruited. Our results obtained in PBMCs, showing a decrease rate of protein synthesis, support the findings of the previously mentioned PET imaging studies and acknowledge PBMCs as a brain’s accessible surrogate model.

## Methods

### Study population and ethics

We performed an exploratory study including participants of a Neurodevelopmental Biobank (BEN; 2020–3616, approved by the Ethical Board of the Institution), specifically: one group of 13 FX and a group of 14 healthy controls, all males matched for age. Participants were recruited through the Fragile X Clinic, at the *CIUSSS de l’Estrie-CHUS* (Sherbrooke, Québec, Canada) between August 2017 and September 2019. FX participants were eligible for inclusion if there were males and had a positive molecular diagnosis of a full mutation for FXS (more than 200 CGG, full methylation). As such, we cannot state that the FX population enrolled in this study is representative of a larger population Informed written consent was obtained from their caregiver in accordance with requirements of the Ethics Review Board of the *CIUSSS de l’Estrie-CHUS*. Since FX participants have moderate to severe intellectual disability, they were not able to fully understand the research project. However, they consent freely to the venous puncture. Control individuals were recruited in order to match FX participants according to age, sex and ethnicity. Written informed consent were directly obtained from the control participants. Venous puncture was performed in the morning between 9 and 11 am. FXS individuals had cognitive evaluations as well, performed by a certified neuropsychologist.

### PBMCs isolation

Blood samples were collected by venipuncture into ACD tubes (acid citric dextrose, BD Vacutainer^®^). All subsequent steps were performed at room temperature. PBMCs were subsequently extracted by density gradient using Ficoll-Paque (Ge Healthcare^®^) following the manufacturer protocol with minor modifications. Briefly, blood samples were centrifuged at 300g for 10 minutes to allow separation and collection of plasma rich platelets (PRP). A volume of PBS (phosphate buffer saline), equal to the volume of plasma collected, was then added to each tube. The blood samples were carefully place onto a layer of Ficoll-Paque (blood:Ficoll-Paque ratio of 4:3) and centrifuged at 500g for 30 minutes. PBMCs were thereafter isolated form the interface and collected by centrifugation (500 g, 10 minutes). Cell pellets were wash twice PBS and counted on a DXH-9000 hematology analyzer (Beckman Coulter^®^).

### Platelets isolation

Platelets were extracted from blood sample collected in 8 mL ACD tubes as previously described [32]. Briefly, PRP was separated from whole blood by centrifugation (300 g, 10 minutes). Afterwards, platelets where sedimented from the PRP (2400 g, 15 minutes) and wash twice with PBS containing 5 mM EDTA and counted on a DXH-9000 hematology analyzer (Beckman Coulter^®^).

### Rate of proteins synthesis rate assay

**Fig 1:**
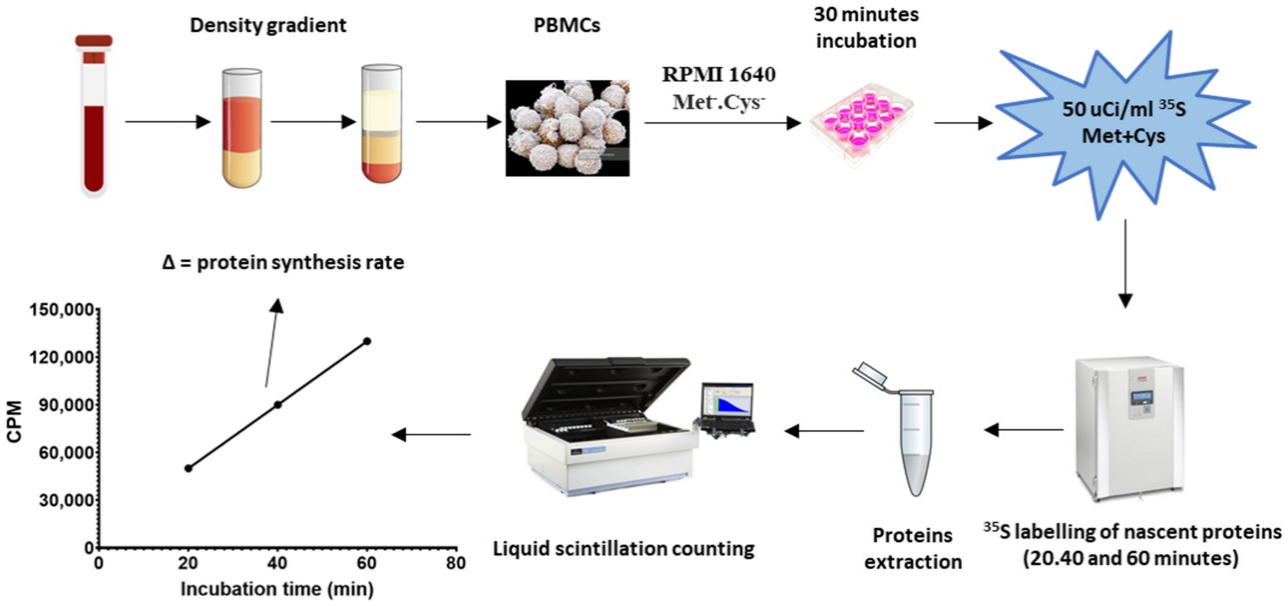
Schematic representation of the rate of protein synthesis assay.

Protein synthesis rate assay is schematised in Fig 1. Freshly extracted PBMCs were suspended into RPMI 1640 (Roswell Park Memorial Institute medium) Met^-^/Cys^-^ (supplemented with 2 mM L-Glutamine, Sigma) and incubated for 30 minutes at 37°C under 5% CO_2_ with gentle agitation. Following methionine and cysteine depletion, 1 million PBMCs were seeded in a well of a 12-well plate containing 0,5 ml of RPMI 1640 Met^-^/Cys^-^ and 50μCi/mL of [^35^S] radiolabeled methionine and cysteine (Perkin Elmer^®^). The cells were then incubated at 37°C (5% CO_2_) under gentle agitation for either 20, 40 or 60 minutes. The latter procedure was performed in triplicate. PBMCs were then pelleted by centrifugation (500 g, 10 minutes) and lysed in PBS containing 2% SDS (sodium dodecyl sulfate). Proteins were precipitated overnight at 4°C in 20% trichloroacetic acid and pelleted by centrifugation (14 000g, 15 minutes). After two washes with acetone, proteins were suspended in 0.1N sodium hydroxide and transferred into a 18ml polyethylene scintillation vial (FisherScientific^®^) containing 7 ml of EcoLite^™^ liquid scintillation cocktail (MP Biomedicals^®^). Each sample was then counted on a LS 6500 liquid scintillation counter (Beckman Coulter^®^) for 1 minute using an energy window of 0–688 keV. Protein synthesis rate was normalised according to cell count and expressed in cpm/min/10^6^ PBMCs. Radiolabeled amino acids incorporation was limited to 60 minutes to ensure unbiased normalisation by cell count (S1 Fig). Experimental conditions including the number of cells, the timeframe and the concentration of radiolabeled amino acids used were rigorously optimised to ensure that the incorporation was linear and provides enough signal strength.

The same assay was used to measure protein synthesis rate in quiescent blood platelets with some minor modifications. After methionine and cysteine depletion, platelets were diluted to 200 million cells/mL and incubated with 250 μCi/mL of [^35^S] radiolabeled methionine and cysteine. Platelets were then incubated under gentle agitation at 37°C under 5% CO_2_ for periods of 30, 60 and 120 minutes (triplicates) and subsequently collected by centrifugation (2400g, 15 minutes). The same protocol as described for PBMCs was used for protein precipitation and liquid scintillation counting. Protein synthesis rate was also normalised to the concentration of cells and expressed in CPM/min/100^6^ platelets.

### Cognitive evaluation

FXS participants had a cognitive evaluation including three different scales validated for the French-Canadian population. First, the Wechsler Adult Intelligence Scale third edition (WAIS-III) was used to evaluate intelligence quotient [35]. Second, the Aberrant Behavior Checklist-Community Edition refactored for FXS (ABC-C_FX_) and the Adaptive Behavior Assessment System (ABAS-II) were administered to assesses aberrant and adaptive behavior respectively [36, 37]. Third, the Behavior Rating Inventory of Executive Function-Adult Version (BRIEF-A) was used to evaluate the executive functions [38].

### Statistical analysis

All results are expressed as means ± standard error of the mean (SEM). An unpaired T-test was used to compare differences in rates of protein synthesis between the two groups. Rate of protein synthesis correlation with clinical parameters were accessed by both Pearson and Spearman correlation. All statistical tests were two-tailed, and a *p* < 0.05 was considered significant. All statistical analysis was conducted in GraphPad Prism (8.4.0)

## Results and discussion

### Population characteristics

The study population include two male groups: 13 FX subjects with a mean age of 28,4 ± 4,6 years and 14 healthy controls, with a mean age of 30,8 ± 7,8 years. All participants were Caucasians. Demographic characteristics of each participant are listed in S1 Table.

### Platelets ^35^S incorporation rate is too low for efficient protein synthesis measurement

**Fig 2:**
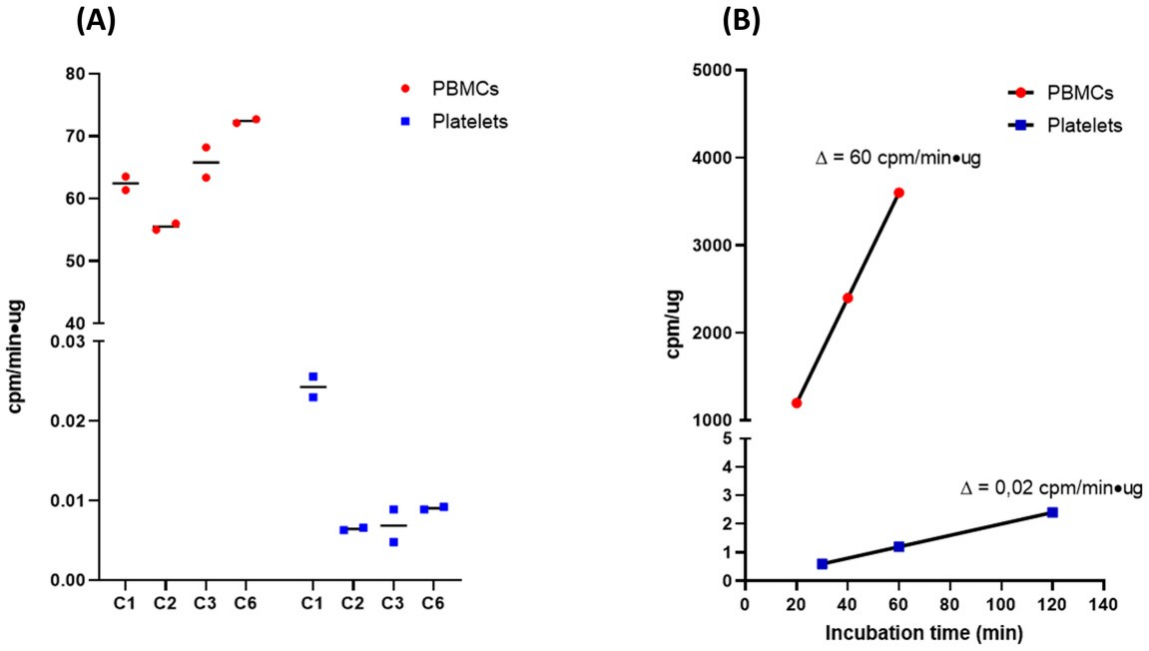
Comparison between rate of protein synthesis measured in PBMCs and platelets. (A) Rate of protein synthesis were compared between PBMCs and platelets of 4 control individuals. Rate of protein synthesis measurement in PBMCs is greater (3 order of magnitude) than in platelets. (B) Typical result obtained from a control PBMCs and platelets. All results were normalised according to protein quantification to ensure correct comparison between both cell types.

The methodology used in this study was based on radiolabelled amino acids incorporation to ensure an unbiased and robust measurement of proteins synthesis [39]. Using this method, we first investigated the relevance of using blood platelets for the monitoring of protein synthesis rate alterations in FXS. Indeed, platelets are one of the most valuable peripheral model for FX due to their close biochemical similarities with neurons and the fact that they replicate some of FXS core molecular alterations [33]. However, the translational machinery of platelets is scarce and devoted to the production of a limited number of proteins during its short lifespan of 10 to 14 days [40, 41]. As expected, even after optimization of the method, the rate of protein synthesis is barely measurable in platelets (Fig 2A and 2B) making them unsuitable for our purpose.

We therefore decided to use PBMCs, which also replicate some of FX core biochemical and molecular defects, as a model to monitor protein metabolism alteration in FXS. Following adjustment for protein content of cells, protein synthesis rate measured in PBMCs is 3 thousand-fold higher than in platelets (Fig 2A and 2B). This observation clearly demonstrates that PBMC’s rate of protein synthesis is sufficiently high to assess differences between FXS and control populations.

### Rate of protein synthesis is decrease in fragile X PBMCs

**Fig 3:**
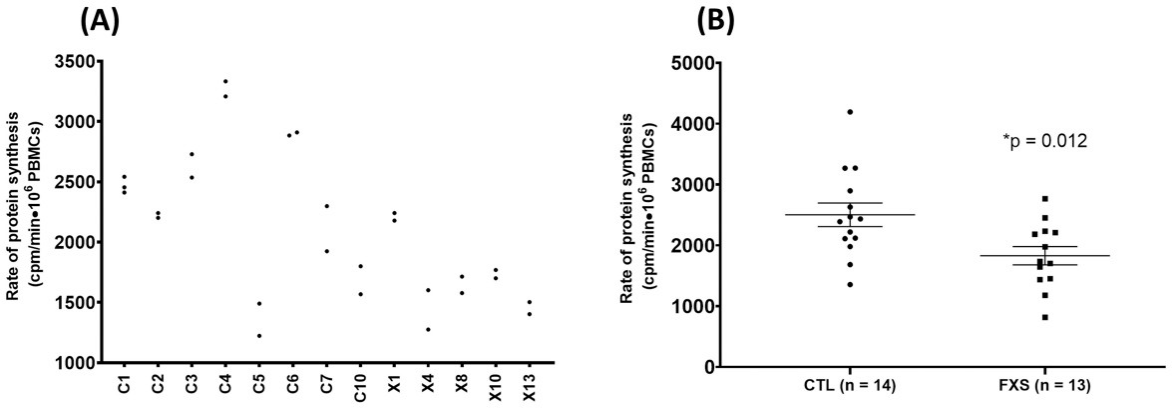
A decreased rate of protein synthesis is observed in fragile X PBMCs. (A) Intraindividual variability of the protein synthesis rate measurements. Each dot indicates a single experiment. (B) PBMCs rates of protein synthesis measurement obtained from 14 controls males and 13 FX males. A 26.9% decrease is observed (mean ± SEM) in the FX group (1830 ± 150 CPM/min) when compare to the control group (2500 ± 194 CPM/min). Error bars represent the SEM. Unpaired T-test.

One of the greatest challenge encounters when undergoing *ex vivo* experiments on human peripheral cells is the individual heterogeneity inherent to the use of such models. The sources of this interindividual variability are highly diverse (genetic background, epigenetic factor, life habits, drugs consumption, etc.) and can easily concealed differences between control and affected cells. A stringent and robust experimental protocol is thus mandatory to limit those fluctuations and ensure results reliability. In concordance with this mindset, we firstly ascertain our assay optimization by investigating intraindividual variability. As illustrated in Fig 3A, our method, using this small subset of data, shows a mean intraindividual variability of 8.6% and a median value of 6.8%, even when measurements were made several months apart (Table 1). This promising reproducibility will obviously facilitate our ability to detect small difference regarding rate of protein synthesis between FX and CTL PBMCs. Therefore, we confidently measured rates of protein synthesis from PBMCs obtained from 13 FX males and 14 matched control individuals. The average rate of protein synthesis was 1830 ± 141 cpm/min for the FX group as compared to 2500 ± 194 cpm/min for the control group (Fig 3B and Table 2). This represent a 26.9% decrease of PBMCs rate of protein synthesis in the FX cohort (*p* = 0.0193). No correlation was found between subject age and rate of protein synthesis in both groups (S2 Fig).

**Table 1:** Intraindividual variability of the rate of protein synthesis measurements.

ID	Mean rate of protein synthesis measurement (cpm/min)	Absolute Difference between replicate	Variation (Difference/Mean)	Days elapsed between replicate
Fragile X patients				
X1	2209	62	2.8%	175
X4	1438	326	22.7%	35
X8	1646	137	8.3%	70
X10	1734	69	4.0%	63
X13	1453	99	6.8%	29
Control individuals				
C1	2469	66	2.7%	4 and 107
C2	2221	40	1.8%	214
C3	2632	194	7.4%	131
C4	3270	125	3.8%	126
C5	1356	268	19.7%	164
C6	2897	26	0.9%	125
C7	2111	374	17.7%	155
C10	1684	231	13.7%	9

**Table 2:** Measurements of the rates of protein synthesis.

ID	Protein synthesis rate (CPM/min)	
	Replicate 1	Replicate 2
Fragile X cohort		
X1	2240	2178
X2	1179	NA
X3	1703	NA
X4	1601	1275
X5	2451	NA
X6	817	NA
X7	2767	NA
X8	1714	1578
X9	1977	NA
X10	1769	1700
X11	2183	NA
X12	2232	NA
X13	1502	NA
X14	1403	1582
Control cohort		
C1	2454	2541
C2	2201	2241
C3	2729	2535
C4	3333	3208
C5	1490	1223
C6	2884	2910
C7	2298	1924
C8	4195	NA
C9	1980	NA
C10	1568	1799
C11	2436	NA
C12	3273	NA
C13	2390	NA
C14	2120	NA

To our knowledge, our results constitute the very first report of a decrease in the rate of protein synthesis in a human peripheral cellular model of FX. As such, the decreased rates of protein synthesis measured in FX PBMCs is in concordance with the only two reports to ever addressed that topic in FX neurons. Indeed, the Beebe Smith group used PET imaging to measure a decreased in [^11^C] leucine integration into nascent brain proteins of FX individuals in two independent studies [24, 25]. This fact, in addition to the promising reproducibility shown by our method, clearly demonstrates the relevance of using PBMCs as a model to investigate translational alterations in FXS.

Our observations are different to those reported in actively dividing cells, namely FX fibroblasts, where an increase in protein synthesis was observed. The translational machinery in such cells is much more active and focused on sustaining the resource-intensive processes of cellular division whereas in non-proliferative cells, protein synthesis is limited and mainly dedicated to cell maintenance. It is therefore conceivable that the absence of FMRP may have opposite effect in cells having contrasting translational processes. Although the rate of protein synthesis is not necessarily correlated to the proliferation status of the cell, we believe that the alterations in the rate of protein synthesis found in FX PBMCs may display with more fidelity the defect found in neurons, which are also terminally differentiated cells. The adverse trend in protein synthesis defects between PBMCs and fibroblasts may also be explained by divergence regarding FMRP function between those two models. Curiously, the two fibroblasts-based studies reported poor reproducibility for the measurements made exclusively in FXS cell lines. Human skin fibroblasts gradually loose their potency to divide during cell passages and rate of protein synthesis may be affected along the way. The large variability regarding rates of protein synthesis measurements obtained with FX fibroblasts could be the signature of distinct effect of FMRP in protein synthesis whether the potency to divide of the cells is still high or at time they get into senescence. Alternatively, the culture conditions used in those two studies may have also influence the translational activity by inducing a metabolic stress on the fibroblasts, a phenomenon that can impact rate of protein synthesis measurement in a FMRP-dependant manner [42].

For those reasons, we believe that the use of a freshly extracted and non-proliferative model will give a better reflection of the protein metabolism found in neurons, which are primary and terminally differentiated cells, than immortalised or long-lasting cultured cellular models.

### Assessment of PBMCs rate of protein synthesis as a biomarker for FXS

**Fig 4:**
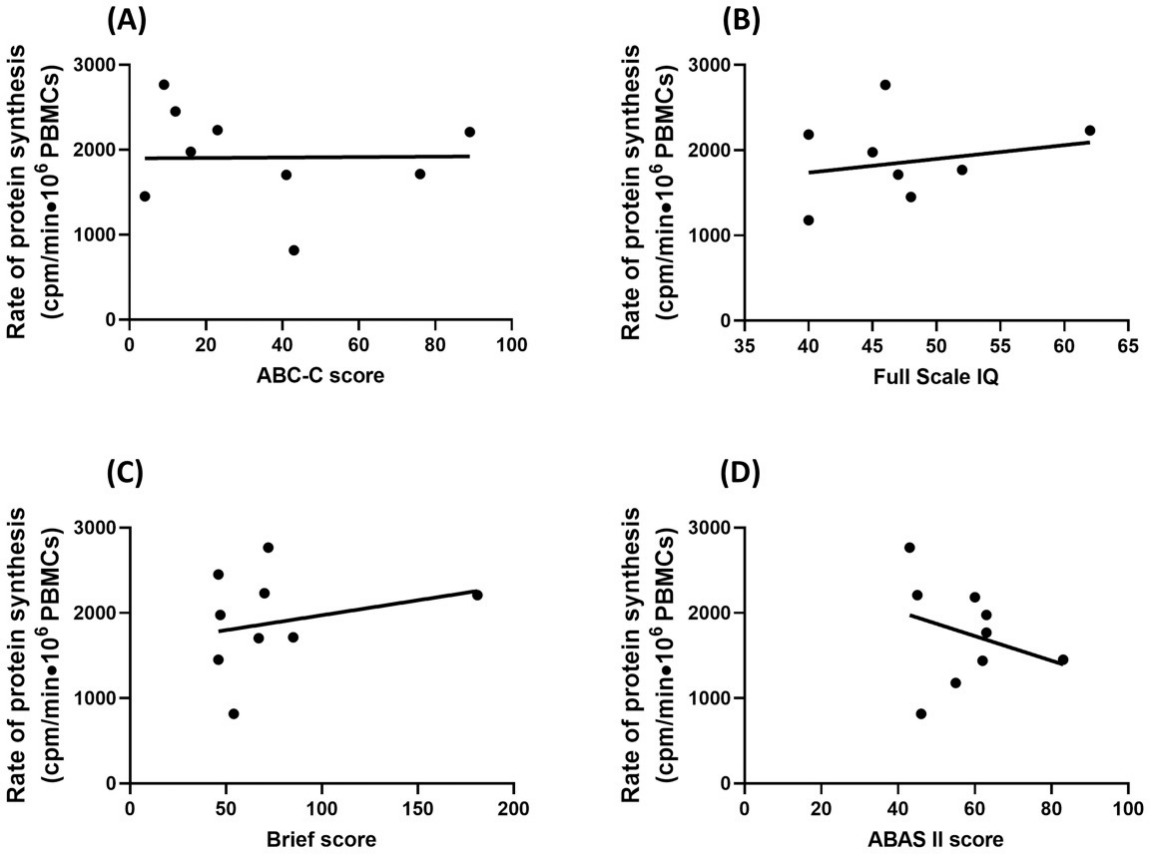
No association between rate of protein synthesis in fragile X individuals PBMCs and cognitive evaluation. (A) ABC-C score (B) Full Scale IQ (C) Brief score and (D) ABAS-II score. No clear correlation is observed.

In order to ascertain the pathophysiological role of protein synthesis in FXS, we evaluated the association between rate of protein synthesis and the clinical phenotype of FX individuals Unfortunately, such correlations were not observed for the cognitive evaluations assessed in this study (Fig 4, Table 3 and S2 Table). Similar results were obtain with a larger cohort of fibroblasts derived from 21 FX males [43].

**Table 3:** Association between rate of protein synthesis measurements and cognitive evaluations.

Variable	Pearson correlation		Spearman correlation	
	r	p	r	p
ABC-C score	-0.195	0.616	-0.250	0.521
Full Scale IQ	0.239	0.569	0.156	0.716
Brief score	0.234	0.545	0.251	0.511
ABAS II score	-0.250	0.517	-0.293	0.442

This lack of association in our study can easily be explained by the study design. Indeed, we purposely recruited fully mutated FX males in order to restrain the heterogeneity of the FX cohort enrolled.; the downside being that all FX subjects presents low cognitive profile. Our strategy allowed us to monitor a significant difference between the FX and control group with a relatively small sample size. On the other hand, this approach did not allow us to get a comprehensive portrait of the translational defects in a sample representative of the entire FX population. As such, our data cannot rule out if the amplitude of the rate of protein synthesis alteration found in FX individuals is related to the severity of the clinical presentation; such observations being out of the scope of the present study.

Clinical trials of FXS are currently limited by the lack of objective tools to assess treatment efficiency in individuals. The development of such monitoring biomarkers could drastically improve the scope of those studies and promotes a better understanding of the human physiopathology [44]. As seen in animal researches, rate of protein synthesis normalisation could be used to address this issue [20, 21]. We truly believe that the method described in this paper is well suited for this task. Indeed, our radiolabeled assay showed a promising intraindividual reproducibility, which mean that it could easily detect improvement in rate of protein synthesis upon treatment. Furthermore, unlike fibroblasts, PBMCs are a well designated model for human trials due to their non-invasive nature, ease of collection and the fact that they replicated the protein synthesis defect found in FX neurons [24, 25]. Nonetheless, the utilisation of this procedure in large multi-center trial might be somewhat limited due to the fact that it must be done in a timely fashion.

## Conclusion

Our work presents the first evidence of a diminution of protein synthesis rate in a human peripheral model of FX, supporting the results obtained from FX brains by PET imaging [24, 25]. These observations highlight the limitations of animal models to fully replicates the complete spectrum of FX phenotypes and that rate of protein synthesis alterations are still a misunderstood hallmark of FXS. This highlight the need of studying human models and subjects to better understand the impact of FMRP’s absence on translation and its contribution to FX physiopathology.

## Supporting information

S1 Fig The number of PBMCs in suspension is stable upon 60 minutes of radiolabeled amino acids incorporation. After extraction from blood sample, the PBMCs were resuspended in RPMI 1640 and incubated for 30 minutes at 37°C under gentle agitation. This step aims to mimic the depletion phase used in the rate of protein synthesis assay. Afterward, PBMCs were diluted to 2 million cells/mL and incubated for up to 120 minutes at 37°C under gentle agitation and the cell were counted (on a flow cytometer) every 30 minutes. PBMCs concentration was stable upon 60 minutes of culture. Afterward. the number of cells in suspension, especially monocytes, rapidly decreased.
(PDF)

S2 Fig No correlation is found between age and protein synthesis rate measurement. In (A) All 27 participants of the study (control and FXS). (B) The control group (n = 14) (C) The Fragile X group (n = 13). (D) No clear correlation is observable in any of those groups.
(PDF)

S1 Table Demographic characteristics of the population. (XLSX)

S2 Table Cognitive evaluations of FX participants. (XLSX)
