# Supplementary material for: Rates of protein synthesis are reduced in peripheral blood mononuclear cells (PBMCs) from fragile X individuals
Source: PLoS One. 2021 May 11;16(5):e0251367. doi: 10.1371/journal.pone.0251367 (PMC8112704; doi:10.1371/journal.pone.0251367)
Supplement: S1 Fig — After extraction from blood sample, the PBMCs were resuspended in RPMI 1640 and incubated for 30 minutes at 37°C under gentle agitation. This step aims to mimic the depletion phase used in the rate of protein synthesis assay. Afterward, PBMCs were diluted to 2 million cells/mL and incubated for up to 120 minutes at 37°C under gentle agitation and the cell were counted (on a flow cytometer) every 30 minutes. PBMCs concentration was stable upon 60 minutes of culture. Afterward. the number of cells in suspension, especially monocytes, rapidly decreased. (PDF) [file pone.0251367.s001.pdf]

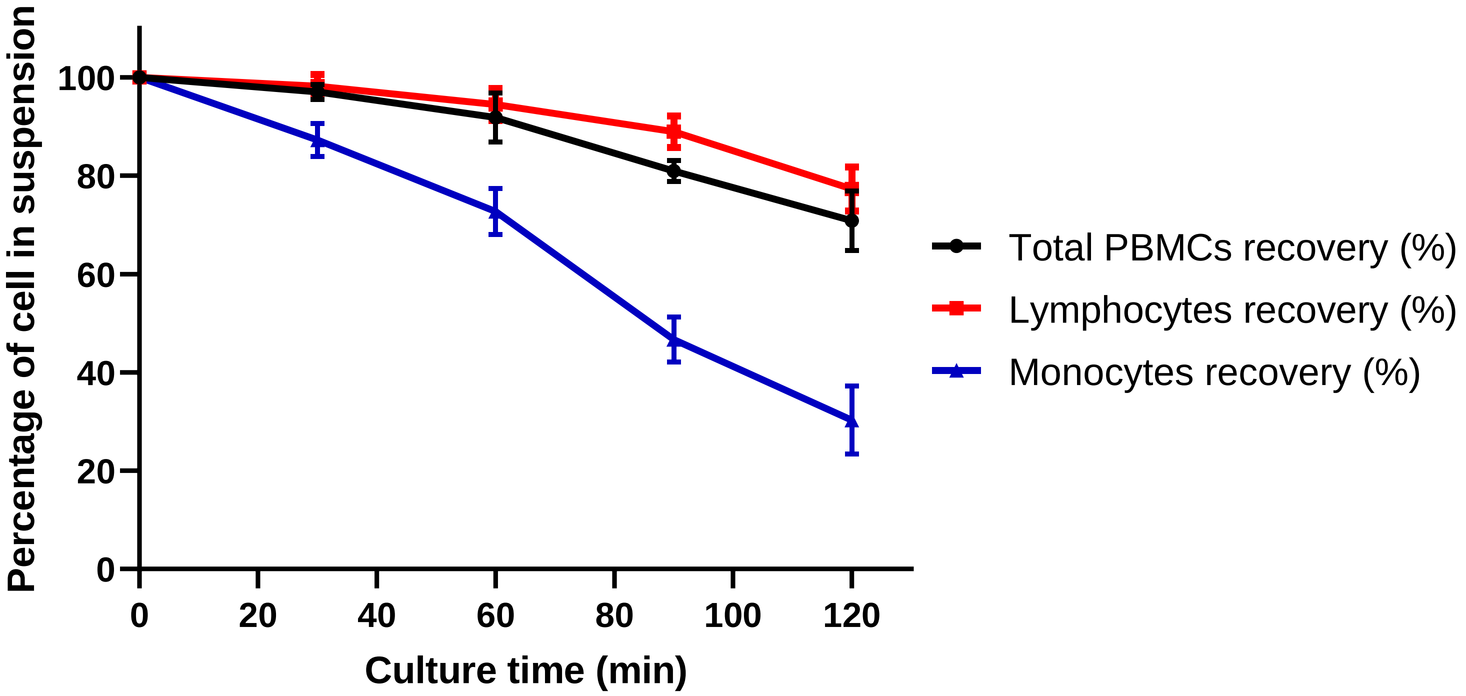

S1 Fig. The number of PBMCs in suspension is stable upon 60 minutes of radiolabeled amino acids incorporation: After extraction from blood sample, the PBMCs were resuspended in RPMI 1640 and incubated for 30 minutes at 37°C under gentle agitation. This step aims to mimic the depletion phase used in the rate of protein synthesis assay. Afterward, PBMCs were diluted to 2 million cells/mL and incubated for up to 120 minutes at 37°C under gentle agitation and the cell were counted (on a flow cytometer) every 30 minutes. PBMCs concentration was stable upon 60 minutes of culture. Afterward. the number of cells in suspension, especially monocytes, rapidly decreased.
