# Supplementary material for: Rates of protein synthesis are reduced in peripheral blood mononuclear cells (PBMCs) from fragile X individuals
Source: PLoS One. 2021 May 11;16(5):e0251367. doi: 10.1371/journal.pone.0251367 (PMC8112704; doi:10.1371/journal.pone.0251367)
Supplement: S2 Fig — In (A) All 27 participants of the study (control and FXS). (B) The control group (n = 14) (C) The Fragile X group (n = 13). (D) No clear correlation is observable in any of those groups. (PDF) [file pone.0251367.s002.pdf]

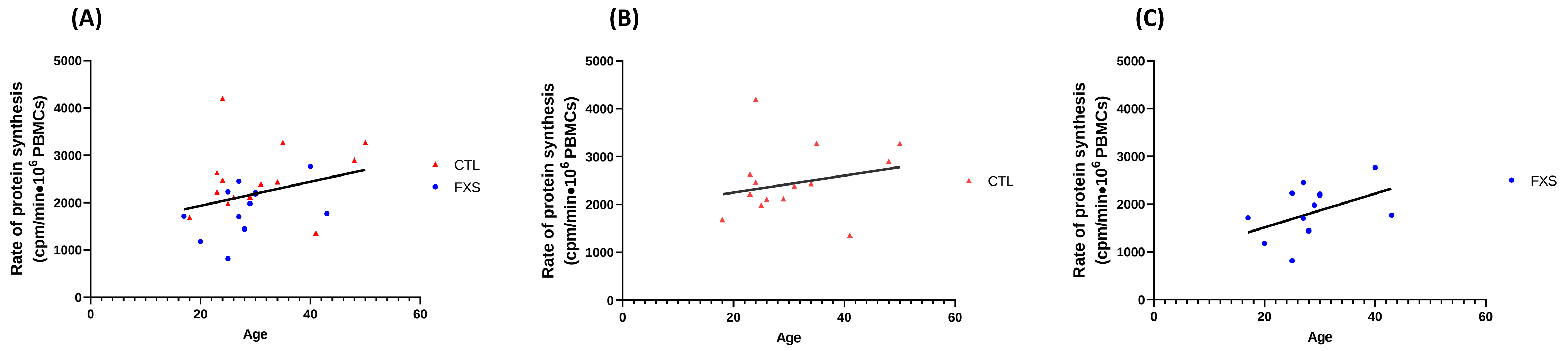

(D)

Group	Pearson correlation		Spearman correlation	
	r	p	r	p
All participants (n = 27)	0.293	0.130	0.232	0.236
CTL (n = 14)	0.393	0.165	0.340	0.233
FXS (n = 13)	0.204	0.484	0.234	0.419

S2 Fig. No correlation is found between age and protein synthesis rate measurement in: (A) All 27 participants of the study (control and FXS). **(B)** The control group (n = 14) **(C)** The Fragile X group (n = 13). No clear correlation is observable in any of those groups **(D)**.
